# Modeling the impact of air, sea, and land travel restrictions supplemented by other interventions on the emergence of a new influenza pandemic virus

**DOI:** 10.1186/1471-2334-12-309

**Published:** 2012-11-19

**Authors:** Ka Chun Chong, Benny Chung Ying Zee

**Affiliations:** 1 Division of Biostatistics, The Jockey Club School of Public Health and Primary Care, The Chinese University of Hong Kong, Hong Kong SAR, China

## Abstract

**Background:**

During the early stages of a new influenza pandemic, travel restriction is an immediate and non-pharmaceutical means of retarding incidence growth. It extends the time frame of effective mitigation, especially when the characteristics of the emerging virus are unknown. In the present study, we used the 2009 influenza A pandemic as a case study to evaluate the impact of regulating air, sea, and land transport. Other government strategies, namely, antivirals and hospitalizations, were also evaluated.

**Methods:**

Hong Kong arrivals from 44 countries via air, sea, and land transports were imported into a discrete stochastic Susceptible, Exposed, Infectious and Recovered (SEIR) host-flow model. The model allowed a number of latent and infectious cases to pass the border, which constitutes a source of local disease transmission. We also modeled antiviral and hospitalization prevention strategies to compare the effectiveness of these control measures. Baseline reproduction rate was estimated from routine surveillance data.

**Results:**

Regarding air travel, the main route connected to the influenza source area should be targeted for travel restrictions; imposing a 99% air travel restriction delayed the epidemic peak by up to two weeks. Once the pandemic was established in China, the strong land connection between Hong Kong and China rendered Hong Kong vulnerable. Antivirals and hospitalization were found to be more effective on attack rate reductions than travel restrictions. Combined strategies (with 99% restriction on all transport modes) deferred the peak for long enough to establish a vaccination program.

**Conclusion:**

The findings will assist policy-makers with decisions on handling similar future pandemics. We also suggest regulating the extent of restriction and the transport mode, once restriction has been deemed necessary for pandemic control. Although travel restrictions have yet to gain social acceptance, they allow time for mitigation response when a new and highly intrusive virus emerges.

## Background

When an emerging influenza virus is introduced to human populations, the pandemic potential of the virus becomes a public concern. Policy makers consider different interventions to contain and mitigate incipient pandemic growth. However, pharmaceutical interventions such as vaccines are not usually available in the early stage of pandemics. Public health measures such as travel restrictions then become essential in controlling pandemic spread.

Novel influenza A (H1N1), also called swine flu, is a novel influenza virus that caused its first illness in Mexico in 2009. Because of insufficient information regarding this particular infectious agent, the World Health Organization (WHO) declared the event the first global H1N1 influenza pandemic (H1N1pdm) on June 11, 2009. In a recent study, an estimated 284,500 deaths have been associated with H1N1pdm [1]. The high transmissibility of the virus has heightened public awareness of disease control measures. Hong Kong’s large-scale international travel pattern and high population density renders the Hong Kong population especially vulnerable. Nearly 300 severe H1N1 cases and 80 fatal H1N1 cases had been reported in Hong Kong at the end of the 2010 flu season [2]. The virus has been widely circulated locally, and lessening the disease burden now depends on implementing effective control measures.

The earliest applied H1N1pdm control measure imposed by the Hong Kong Government was travel restriction via travel advice and entry screening [3]. For highly-transmittable infectious diseases such as influenza, the traveling patterns of individuals play an essential role in geographical disease spread. Travel restrictions, a type of social control measure, have been evaluated in several epidemics including influenza [4-7], human immunodeficiency virus (HIV) [8], severe acute respiratory syndrome (SARS) [6,9], and, recently, the 2009 H1N1pdm [10]. Empirical statistics indicate that the influenza season was delayed following reduced flying activity caused by the US 9/11 incident [11]. Hufnagel *et al*. [9] further demonstrated that isolating a mere 2% of the largest cities was enough to halt the SARS outbreak. Nevertheless, the WHO considers travel restriction to be impractical in the majority of countries [12]. In addition, some studies have disputed the value of air travel restrictions in epidemic control [6,7,13]. Cooper *et al*. regarded that benefits accrued from suspending air travels is limited by the short serial interval of influenza. Hollingsworth *et al*. [6] concluded that containment of a pandemic influenza strain requires rigorous travel restrictions and small numbers of local infectious inhabitants. In Hong Kong, because the magnitude of travel restrictions imposed by travel advice and entry screening was small, its effectiveness in pandemic delay is disputable.

**Figure 1:**
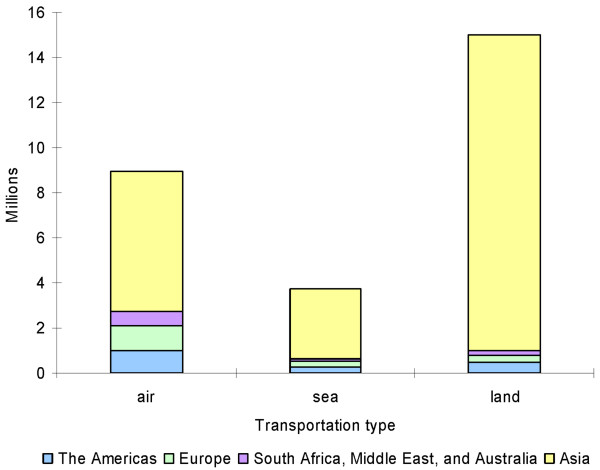
**Total arrival (in millions) by air, sea, and land transport.** Forty-four countries were selected in total which contributed more than 95% of arrivals to Hong Kong.

Despite these limitations, the impact of travel restrictions requires ongoing investigation. Previous studies focused on air travel restrictions alone [4,14], but in many cities, including Hong Kong, air travel is a secondary means of transport for arriving and departing travelers. Statistics show that more than half of the passengers arriving in Hong Kong annually enter by sea or land [15]. As shown in Figure 1, over ten million visitors per annum enter Hong Kong via land transport from Asia. Visitors from North America and Europe constitute a relatively high proportion of air transport arrivals. Therefore, to assess the true effectiveness of travel restrictions, air, sea, and land transport must all be incorporated into the evaluation. Additionally, most of the published mathematical models admit only those latent individuals who travel between countries. However, with limited screening sensitivity at the entry border points [16,17], a large number of infected cases could enter, thereby dramatically increasing the rate of local disease transmission [18].

Whereas travel restrictions can be imposed almost immediately, antiviral drugs require extended time for preparation. In Hong Kong, antiviral and hospitalization strategies were implemented about 3.5 months after the first global H1N1pdm import [19]. The main purpose of travel restrictions are to defer the pandemic, whereas antivirals and hospitalizations aim to reduce the transmission rate and severity of disease [5]. These strategies have proven useful in many influenza epidemics, including that of the novel H1N1pdm. Vaccination alone effectively mitigates most of the epidemics, by reducing the risk of a susceptible being infected, and thus the possibility of seeding the disease in the community. Nevertheless, vaccine design, development and public administration are lengthy processes. Current manufacturing capacity is insufficient to produce the vaccines within a few months following declaration of an influenza pandemic [20]. Hong Kong Government officials implemented the H1N1 vaccination program about nine months following the first global import [21], by which time the H1N1pdm had passed its peak. Low public acceptance of vaccine uptake during the H1N1pdm period compounded the issue. In one study, only 7% of subjects reported that they were ‘likely/very likely/certain’ to be vaccinated [22].

Impact of epidemic interventions is usually quantified by mathematical models. Clinical trial design is impractical for assessing the effectiveness of some interventions, such as face masks and isolation, because of ethical considerations relating to epidemics in general. By using mathematical models, the epidemic dynamics and intervention effectiveness can be determined. Such models can evaluate a range of interventions; isolation [23], quarantine [24], antiviral drugs [25,26], school closures [27,28], vaccinations [29,30] and face masks [31], among others.

In the study, we use the Hong Kong Governmental response to the 2009 H1N1pdm as a model case study to evaluate the effectiveness of travel restrictions of different magnitudes and transport modes i.e. air, sea, and land, combined with other interventions, namely antivirals and hospitalizations, in the event of a novel influenza virus. The impact is assessed by simulations from an epidemic model. We also investigate the effects of changing important parameters, including reproduction numbers in non-local visitors to Hong Kong, screening sensitivity at entry border points, and date at which travel restrictions are imposed. The results provide valuable information to policy-makers and public health experts in the event of similar future pandemics.

## Methods

### Population and transportation

Population data were extracted from the International Database (IDB), U.S. Census Bureau [32]. The individual probability of travel for each country was calculated as the daily travel rate divided by the population size. The arrival data were extracted from visitor arrival statistics provided by the Hong Kong Tourism Board [15]. These statistics include the total number of arrivals by country, together with their modes of transport. Forty-four countries, collectively contributing more than 95% of annual arrivals to Hong Kong, were selected for the analysis (Figure 1). The yearly frequency of departing Hong Kong residents by different transport modes were collected from the Census and Statistics Department, Hong Kong [33]. The data are listed in Additional file 1 and are assumed to be uniformly distributed on a daily scale.

### Disease transmission model

We extended the discrete stochastic *SEIR* model [34-36] to study the H1N1pdm dynamics and the impacts of local interventions. The stochastic approach differs from that of deterministic models [4,37-41]. In our model, foreign virus arriving by air, land, and sea transport adapts and establishes in a local community with inherent uncertainty. Introducing this chance effect into the epidemic dynamics enhances the realism of the model. The model outputs are the deferred time period and the illness attack rate (AR) (defined as the number of new infected cases per head of population during a given time period).

All individuals in the local population were assumed to be susceptible, and the average latent and infectious periods were set to 1.45 and 2.9 days, respectively [10,42]. The population, *N*, was divided into four classes: susceptible (*S*(*t*)); exposed (*E*(*t*)); infectious (*I*(*t*)); and recovered (*R*(*t*)), at each time point *t*. Because no information was available on cross-immunity from past influenza infections, the initial population was set at 100% susceptible. Once susceptible individuals became infected, they advanced to the latent (non-infectious) stage. Following the latent period, they became infectious and could transmit the disease to other susceptible individuals. A number of individuals moved to the next compartment with a defined probability. The number of individuals advancing to each stage was assumed to follow a binomial distribution.

#### Disease transmission from travelers

In the disease transmission model, latent ( $\left(IM_{k}^{E}(t)\right)$) and infectious ( $\left(IM_{k}^{I}(t)\right)$) travelers arrived from 44 foreign countries by transport *k*-th and were assigned to compartments *E*(*t*) and *I*(*t*), respectively. Because the screening sensitivity at the border points of entry was limited, a proportion (1−*ν*) of infectious cases were imported to Hong Kong, an approach not considered in other global patch models [7,10]. The number of cases imported to the local city was also assumed to be binomially distributed, with a probability equal to the chance of travel via the specified transport mode.

To allow for spatial heterogeneities between non-local countries, case numbers for each country were generated from discrete time SEIR models assigned independent reproduction numbers (*R*_0_), defined as the average number of secondary infections induced by a typical infectious individual in a wholly susceptible population. The magnitude of *R*_0_depends on the individual contact rate, disease transmissibility, and the duration of infectiousness; hence, *R*_0_ is expected to differ between countries. In this paper, the *R*_0_of foreign countries were estimated by the initial exponential growth rate method [43] assuming no intervention during the early stage of H1N1pdm. They were fitted by daily counts of laboratory-confirmed infected cases in each country, obtained from pandemic H1N1 situation updates archived in the World Health Organization (WHO) [44] and the European Centre for Disease Prevention and Control (ECDC) [45]. Several local exposed (*EX*^*E*^(*t*)) and infectious (*EX*^*I*^(*t*)) cases were removed from the compartments based on the proportion of travel by the specified means of transport. Simulation was started from the day of initial global import. The effect of varying the *R*_0_s of specified foreign countries by 20% was performed. The details of disease transmission from travelers are provided in Additional file 1.

#### Control measures

The mathematical model assesses the effectiveness of: (i) travel restrictions (for different transport modes) and (ii) local antiviral and hospitalization interventions. Travel restrictions were supposed to take effect on the day following the first global onset case. Different start dates were tested in a sensitivity analysis. The antiviral and the hospitalization strategies were implemented locally 3.5 months following the first global onset case, echoing the strategies employed by the Department of Health, Hong Kong [19].

##### Travel restrictions relating to sea, land, and air travel

We imposed 90% and 99% travel restrictions (*f*_*k*_), on different transport modes *k*. The term ‘travel restriction by *f*_*k*_’ meant allowing only a fraction of (1−*f*_*k*_) import individuals to be transported to Hong Kong from overseas through transportation *k*. We also considered only one-third (*ν*) of those (1−*f*_*k*_) imported infectious cases as successfully identified positive cases at the entry borders in the baseline scenario [16]. The screened positive individuals entering Hong Kong were transported to hospital for further examination [3]. Confirmed cases were recommended to undertake voluntary quarantine. We assumed that all identified cases accepted voluntary quarantine. Screening sensitivities of 95% and 5%, and travel-restriction start dates of three and five months following the first global import, were also evaluated (Additional file 1).

##### Antiviral and hospitalization

We assumed that 12% (*p*_*T*_) of the infectious subjects were offered antiviral and 6% (*p*_*H*_) of the infectious subjects were hospitalized, based on influenza pandemic records [23,25]. The remaining 82% (*p*_*U*_) of infectious individuals were untreated. The antiviral reduces infectiousness (*ψ*) of individuals by 60% [46]. Either intervention reduce the average infectious period by 1.5 days [47]. Compartments for antiviral *T*(*t*) and hospitalization *H*(*t*) were developed separately in the model for individual assessment of the treatments. The stochastic system is

(1) $$\begin{array}{l} S(t+\mathrm{\Delta t})=S(t)-B(t)\\ E(t+\mathrm{\Delta t})=E(t)+B(t)\\ \;+\underset{k}{\sum }(1-f_{k})IM_{k}^{E}(t)-EX^{E}(t)-C(t)\\ I(t+\mathrm{\Delta t})=I(t)+C(t)+(1-\nu )\underset{k}{\sum }(1-f_{k})IM_{k}^{I}(t)\\ \;-EX^{I}(t)-D(t)-M(t)-N(t)\\ T(t+\mathrm{\Delta t})=T(t)+M(t)-P(t)\\ H(t+\mathrm{\Delta t})=H(t)+N(t)-Q(t)\\ R(t+\mathrm{\Delta t})=R(t)+D(t)+P(t)+Q(t) \end{array}$$

We denote *bin*(*m, n*) as a binomial distribution with probability *m* and number of total individuals *n*. The distributions of the classes are

(2) $$\begin{array}{l} B(t)∼\text{bin}(1-\exp[-\frac{\beta }{N}[I(t)+(1-\psi )T(t)\\ \;+H(t)]\mathrm{\Delta t}]),S(t))\\ C(t)∼\text{bin}(1-\text{exp}(-\mathrm{\alpha \Delta t}),E(t))\\ M(t)∼\text{bin}(p_{T}\mathrm{\Delta t},I(t))\\ N(t)∼\text{bin}(p_{H}\mathrm{\Delta t},I(t))\\ D(t)∼\text{bin}(p_{U}[1-\text{exp}(-\gamma_{R}\mathrm{\Delta t})],I(t))\\ P(t)∼\text{bin}(1-\exp(-\gamma_{T}\mathrm{\Delta t}),T(t))\\ Q(t)∼\text{bin}(1-\text{exp}(-\gamma_{H}\mathrm{\Delta t}),H(t)) \end{array}$$

where *β* is the disease transmission rate and 1/*α*is the average latent period. The probability of a susceptible person becoming infected is 1−*exp*[−*β*[*I*(*t*) + (1−*ψ*)*T*(*t*) + *H*(*t*)]/*N*] during time step *Δt*. *γ*_*R*_, *γ*_*T*_, and *γ*_*H*_ specify the removal rates from the infectious state, the antiviral treatment state, and the hospitalization state, respectively. The details of the mathematical methodology and the simulation are provided in Additional file 1.

### Epidemic evolution and baseline scenario

The H1N1pdm is seeded according to the start dates of each country (listed in Additional file 1). The earliest epidemic was seeded in Mexico on March 11, 2009 [48]. For each country, the number of infected cases was generated from the discrete-time *SEIR* model, based on the estimated reproduction number.

Since the Hong Kong Government confirmed the first imported case of H1N1pdm on May 1, 2009 [49], the initial numbers of latent cases and infectious cases were iteratively estimated, thereby minimizing the difference between the reported date and the simulated first passage time (FPT). Allowing for stochastic variability, the baseline transmission rate was estimated for the first two months following the day of the first local import, in the absence of travel restrictions and intervention. Local daily surveillance of confirmed infected cases (May 1, 2009 to June 30, 2009) was available from press releases on human swine flu, published by the Department of Health, Hong Kong [50]. The range of *R*_0_ values encompassed mild and severe scenarios.

## Results

### Scenarios with no interventions

**Figure 2:**
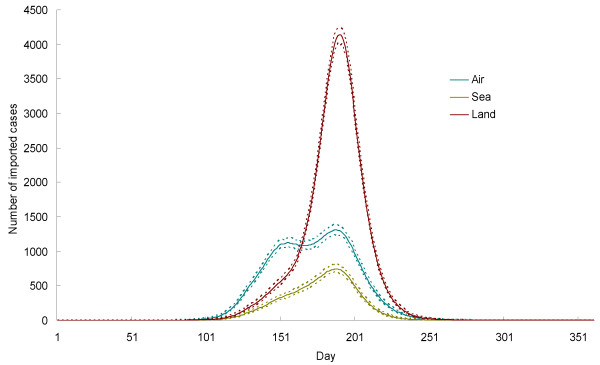
**Number of imported cases to Hong Kong by different transports vs. days with no travel restriction.** Day one was taken to be March 11, 2009 (the time of the first global case onset). The solid lines represent the average cases; the dotted lines represent the corresponding lower and upper bounds of the 95% non-parametric confidence intervals.

The local baseline *R*_0_ was estimated at around 1.4. Values of *R*_0_ were chosen to simulate mild (*R*_0_=1*.*1) and severe (*R*_0_=1*.*7) influenza outbreaks in Hong Kong, and were consistent with those reported in previous studies [48,51]. In foreign countries, *R*_0_ ranged from 1.1 to 1.9. In the baseline scenario (*R*_0_=1*.*4), the median FPT and first one hundred passage time (FHPT) of infected cases entering Hong Kong were 55 and 90 days, respectively (Table 1). Because the H1N1pdm was initiated in the Americas, the primary means by which the infected cases arrived in Hong Kong during the fourth month was air travel (Figure 2). The number of cases imported by air transport exceeded that of land transport during the first six months. Thereafter, because the emerging virus had circulated to most of the Asian countries, including China, the number of cases imported by land transport increased exponentially. Because ships constitute a minor transport mode to Hong Kong, they delivered few cases throughout the pandemic period (Figure 2).

**Table 1:** Median FPTs and FHPTs (in days) with confidence intervals (CI) at the baseline scenario

**Control measure**	**Transportation**	**FPT (95% CI)**	**FHPT (95% CI)**
No travel restriction		55 (35, 67)	90 (89, 92)
90% travel restriction	Air	62 (42, 72)	99 (97, 100)
	Sea	56 (34, 67)	92 (90, 93)
	Land	58 (44, 69)	93 (91, 95)
	Air, Sea	66 (51, 77)	102 (101, 104)
	Air, Land	69 (45, 81)	106 (104, 107)
	Sea, Land	58 (30, 69)	95 (93, 96)
	All transports	94 (88, 98)	114 (114, 115)
99% travel restriction	Air	61 (37, 72)	99 (97,101)
	Sea	57 (28, 68)	92 (90, 94)
	Land	59 (38, 69)	93 (92, 95)
	Air, Sea	65 (39, 78)	104 (101, 105)
	Air, Land	68 (49, 82)	107 (108, 110)
	Sea, Land	59 (34, 70)	95 (93, 96)
	All transports	117 (116, 118)	138 (138, 139)

Travel restrictions took effect on the day after the first global case onset. The medians and the non-parametric 95% confidence intervals were obtained from 100 simulation runs.

**Figure 3:**
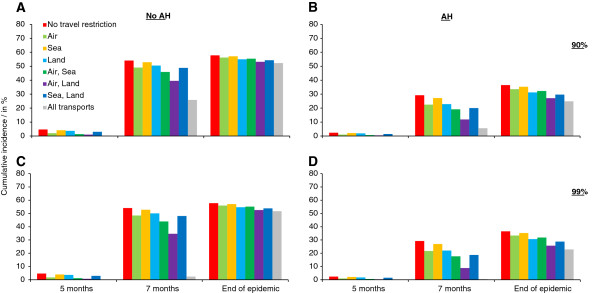
**Median cumulative attack rates (in %) for different control measures at the baseline scenario.** The absences and the presences of the uses of antiviral and hospitalization are illustrated in the left-hand column (**A** and **C**) and in the right-hand column (**B** and **D**), respectively. The upper panel (**A** and **B**) and the lower panel (**C** and **D**) illustrate the 90% and the 99% restriction rescaling, respectively. The medians were obtained from 100 simulation runs; AH = antiviral and hospitalization.

**Figure 4:**
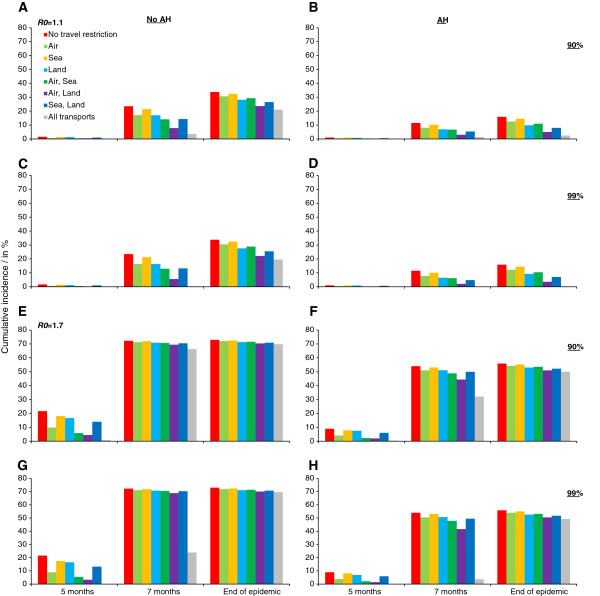
**Median cumulative attack rates (in %) for different control measures at the mild and the severe scenarios.** The absences and the presences of the uses of the antiviral and hospitalization are illustrated in the left-hand column (**A**, **C**, **E**, and **G**) and in the right-hand column (**B**, **D**, **F**, and **H**), respectively. The first and the third panels (**A**, **B**, **E**, and **F**), and the second and the forth panels (**C**, **D**, **G**, and **H**) illustrate the 90% and the 99% restriction re-scalings, respectively. The medians were obtained from 100 simulation runs; AH = antiviral and hospitalization.

In the absence of control measures, and setting Hong Kong *R*_0_=1*.*4, the seven months’ cumulative AR was close to that of the final AR (Figure 3A). In a mild local scenario (*R*_0_=1*.*1), the cumulative AR was a mere 2% after five months, and after seven months, had reached just two-thirds the final cumulative AR (Figure 4A). In a severe local scenario (*R*_0_=1*.*7), the H1N1pdm had nearly terminated after seven months, and the cumulative AR exceeded 70% (Figure 4E).

### Impact of the interventions

**Figure 5:**
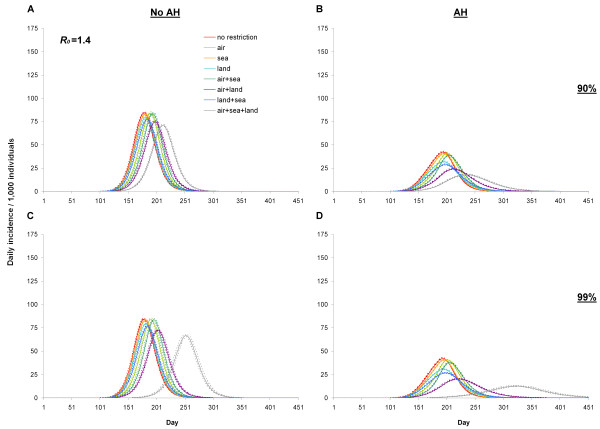
**Daily incidences for different control measures vs. days at the baseline scenario (*R***_***0***_***=1.4*).** The absences and the presences of the uses of antiviral and hospitalization are illustrated in the left-hand column (**A** and **C**) and in the right-hand column (**B** and **D**), respectively. The upper panel (**A** and **B**) and the lower panel (**C** and **D**) illustrate the 90% and the 99% restriction rescaling, respectively. Day one was taken to be March 11, 2009 (the time of the first global case onset). The solid lines represent the average cases; the dotted lines represent the corresponding lower and upper bounds of the 95% non-parametric confidence intervals; AH = antiviral and hospitalization.

Among the three kinds of transport, disease spread was most effectively delayed by restriction on air travel. Air travel restriction delayed the FPT and FHPT by one week relative to the no-intervention control case (Table 1). The peak time might have been delayed by two weeks if a single 99% air travel restriction had been imposed (Figure 5C). The pandemic established in China six months following the first global import to Hong Kong; the strong land connection between the two countries significantly enhanced the number of imported cases. Therefore, suspending both air and land transport could delay the passage time by a further one to two weeks, and the peak by about 3.5 weeks (Figure 5A and C).

Travel restrictions on all transport modes most effectively delayed the spread of the H1N1pdm. As shown in Figure 5, the difference between 90% and 99% transport reduction was apparent only when all three transport modes were restricted. Once the volume of all transports was reduced by 90%, FPT and FHPT were retarded by one month relative to the control case. 99% travel restriction delayed the FPT and FHPT by an additional two months (Table 1). 90% and 99% restriction of all transport modes deferred the peak for about six weeks (Figure 5A), and 12 weeks (Figure 5C), respectively.

Nevertheless, blocking of sea or land transport alone cannot prevent disease spread; it did not confer any large reduction in the five and seven months’ cumulative ARs. Even with sea transports reduced by 99%, the peak is delayed by only one week, relative to the control case (Figure 5C).

In reducing attack rate, antiviral and hospitalization administration (AH) proved more promising than travel restrictions. Neither 90% nor 99% travel restrictions reduced the epidemic magnitude by more than 10%. Implementation of AH on a proportion of infected individuals could halve the peak rate, and reduce the final cumulative ARs (relative to the case of no intervention) from 58% to 37% (Figure 5B and Figure 3B). However, the peak time of epidemic was only slightly delayed.

Combining travel restrictions with AH, the impacts on mitigation are greatly enhanced. Air travel restrictions plus AH delayed the peak time by more than three weeks (Figure 5B and D). A 99% restriction of both air and land travel delayed the peak time by more than six weeks (Figure 5D). Imposing AH plus a 99% restriction on all transport modes flattened the epidemic curve more effectively than did AH plus 90% travel restriction. This strict condition greatly repressed the cumulative ARs, limiting them to around 1% (Figure 3D). Most importantly, the peak was delayed by approximately five months (Figure 5D). Supplemented by AH, total travel restriction reduced the final cumulative AR to about 14%.

**Figure 6:**
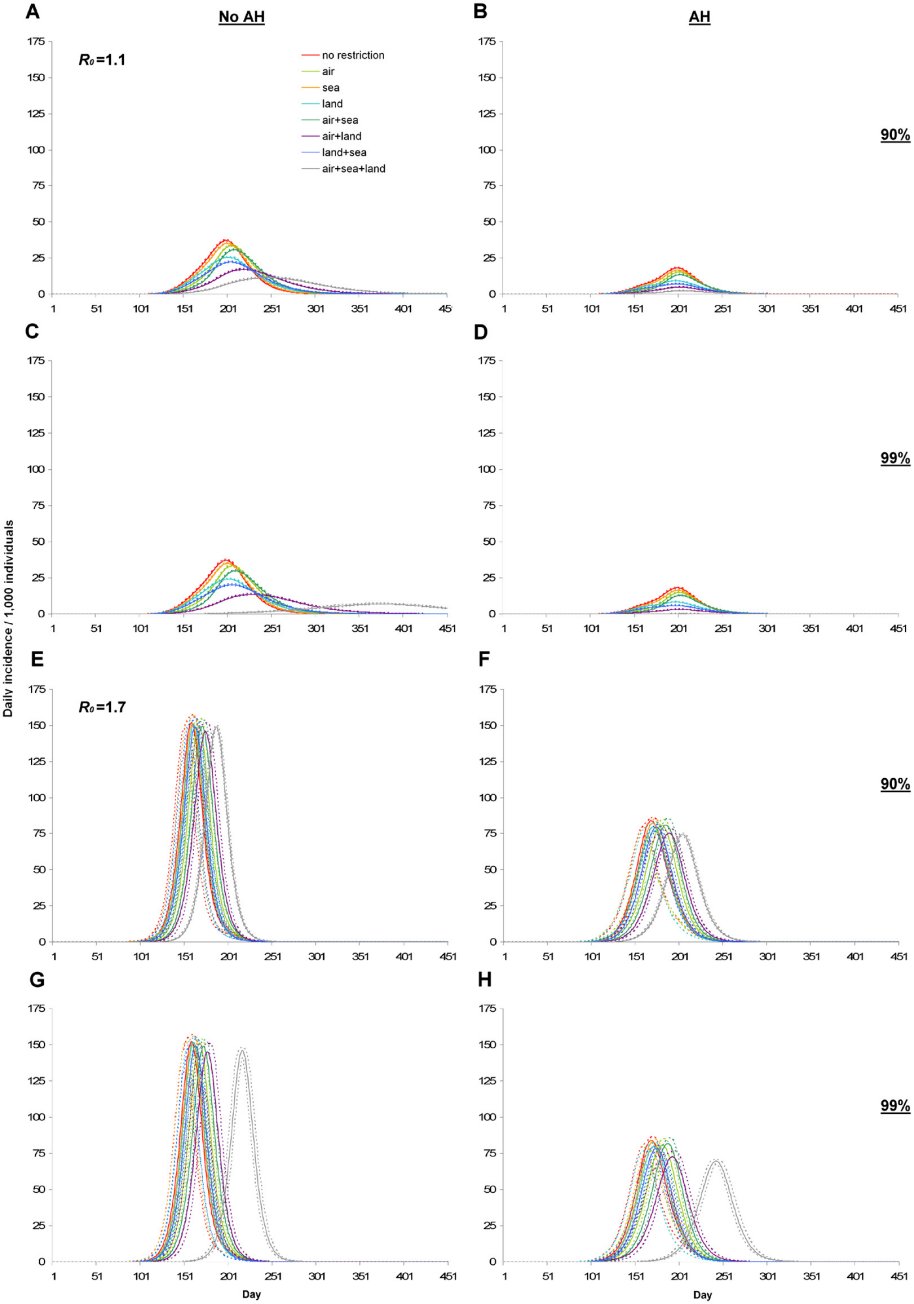
**Daily incidences for different control measures vs. days for the mild (*R***_***0***_***=1.1*) and the severe (*R***_***0***_***=1.7*) scenarios.** The absences and the presences of the uses of the antiviral and hospitalization are illustrated in the left-hand column (**A**, **C**, **E**, and **G**) and in the right-hand column (**B**, **D**, **F**, and **H**), respectively. The first and the third panels (**A**, **B**, **E**, and **F**), and the second and the forth panels (**C**, **D**, **G**, and **H**) illustrate the 90% and the 99% restriction re-scalings, respectively. Day one was taken to be March 11, 2009 (the time of the first global case onset). The solid lines represent the average cases; the dotted lines represent the corresponding lower and upper bounds of the 95% non-parametric confidence intervals; AH = antiviral and hospitalization.

In a milder local scenario (*R*_0_=1*.*1), travel restrictions not only effectively delayed the H1N1pdm, but also flattened the incidence curve. Suspension of air travel remained the best choice among the three transport modes for repressing the cumulative ARs (Figure 4A and C). Because the disease transmissions were comparatively slow and mild, 90% land import restriction was sufficient to decrease the peak ARs by one-third (Figure 6A and C). Besides reducing the peak incidence by 25%, 99% restriction of all transport delayed the peak time by one year following the first global import. As shown in Figure 6B and D, combining AH and travel restrictions resulted in significant peak reduction. Restricting all travel routes as well as administering AH, the spread of the local epidemic was halted; the 99% travel restriction retained the final cumulative AR at around 0.2% (Figure 4B and D).

Incoming travel restrictions became less effective (especially where ARs was concerned) as the contagion level of the influenza virus increased to *R*_0_ = 1*.*7. The rapid disease transmission rate raised the five months’ cumulative AR to an average of 22% (Figure 4E and G). Imposing 99% restriction on all transport modes remained sufficient to retard the disease spread, deferring the epidemic peak time by about eight weeks (Figure 6G). However, under 90% total travel restriction, no significant delay was observed. Supplementation by AH became more important in this scenario (Figure 4F). Because the incidence growth was now suppressed by AH, travel restrictions more effectively repressed the epidemic. Imposing a 99% restriction on all transport, the seven months’ cumulative AR was restrained to 4% or less (on average; Figure 4H), with an approximate delay in peak time of 12 weeks (Figure 6H).

#### Effect of *R*_0_from non-local countries

In our study, we varied the *R*_0_s from 44 foreign countries by 20%, and re-evaluated the model outputs. The changes in foreign *R*_0_s affected the number of imported cases, implying that growth of a local epidemic depends upon the passage times of the cases. With reductions of *R*_0_s, imposing restrictions solely on air travel nearly halved the cumulative ARs. A 99% all-transport restriction was sufficient to halt the local spread (cumulative ARs attain < 0.1%) after seven months (with or without AH administration). It maintained the seven months’ cumulative AR at around 12% even with a 20% increase in *R*_0_.

#### Effect of screening sensitivity at entry border points

Increasing the screening sensitivity at the entry border slightly retards the local epidemic. In most of the travel restriction scenarios, the additional FHPTs delay imposed by the strict 95% screening sensitivity and the relaxed 5% screening sensitivity was, at most, one to two weeks.

#### Effect of implementation date on travel restrictions

Imposing travel restrictions five months after the arrival of the first global case is ineffective. Even with a total transport reduction of 99%, the reduction in the cumulative ARs was found to be negligibly small. By comparison, allowing a three-month gap between arrival of the first global case and imposition of travel restrictions, the seven-months cumulative AR could be restrained at around 2% by imposing AH plus 99% restriction on all transport modes.

## Discussion

Non-pharmaceutical interventions such as travel restrictions are immediate means by which to slow pandemic growth and to extend the time available for vaccine production. Here we collected statistics on arrival numbers in Hong Kong from 44 countries via air, sea, and land transport [15]. These data were input to a mathematical model to evaluate the impact of travel restriction on different scales and by different modes, combined with other government strategies (namely, antivirals and hospitalizations), using the 2009 H1N1pdm as an example. From our results, we infer that the main connecting route and transport mode between source and destination (in this instance, air travel from the Americas/Mexico to Hong Kong), should be targeted for travel restrictions in a pandemic. This is in addition to suspending travels from large, high-density cities [9]. The emerging 2009 H1N1pdm virus had circulated to most Asian countries, including densely-populated China, six months after the first global case was reported. The number of imported cases from China to Hong Kong by land transport thereafter increased exponentially. Reducing land travel could have significantly lowered the number of import transmissions. In mild cases, such a restriction reduces the proportion of peak incidence and delays the peak time by up to one month. However, suspending travels on a single route only slightly decreases the peak incidence and the final epidemic size. Restricting either sea or land transport, but not both, confers little advantage in terms of disease spread.

Travel restrictions may not be effective at reducing epidemic size. Based on our results, antivirals and hospitalization lower the disease incidence as well as the final epidemic size, but do not prevent the import of contagious cases or delay the peak time. In most scenarios, imposing AH on a proportion of infected individuals (< 20%) moderately mitigates the severity of the pandemic, reducing the peak incidence by half. Several previous studies have lauded AH as an effective new epidemic control measure [5,24,52]. On the other hand, when AH and travel restrictions are imposed together they supplement each other, further mitigating the pandemic. Since imposing AH suppresses the growth of local transmission, the number of local infected sources is reduced, while travel restrictions prevent the import of fresh infectious sources. Imposing both interventions thus considerably extends the peak time. When rigorous restriction on all transport modes is combined with AH, the delays (peak appearing after the 10th month) are possible to allow vaccine production (i.e. beyond the nine months following the first global import to Hong Kong, during which time a vaccine program was developed and administered to the local public).

The effectiveness of travel reductions depends upon the rate of epidemic growth in different foreign countries [6]. If control measures had been responsible for reduced transmission in foreign countries (modeled by decreasing the *R*_0_s by an average of 20%), a 99% restriction on all external transport modes might have halted the local spread. In any case, increasing the screening sensitivity at the entry border points conferred a one to two week delay benefit. In reality, some individuals would refuse to undertake voluntary quarantine despite screening positive at the border. Such refusals would decrease the sensitivity for screening of quarantined symptomatic cases. Although the true screening sensitivity may not match our model settings i.e. 30%, we showed that screening sensitivity exerts only a secondary effect on epidemic delay. In the simulation results, the average maximum number of the screened import cases is 928 (95% confidence interval: 895-961), whereas there are 1400 isolation beds in 14 major hospitals in Hong Kong, which was set by the government after SARS [53]. Thus, the control measure would unlikely entail a capacity problem in Hong Kong. Our findings also imply that restrictions be imposed no later than three months following the first infectious global import. Implementing travel restrictions at or beyond the end of the fifth month would be almost useless, because the local epidemic would by then have evolved to a mature stage, in which disease transmission would depend on the local exponential increase in cases, rather than on successive imports.

In the study, we focused on a major city, Hong Kong, as a high-density, well-traveled region especially suited to the assessment of travel restrictions. Travel restrictions reduced the illness rate only in the event of mild local disease transmission intensity. In some rural areas or island countries, the disease transmission intensities as well as the reproduction numbers remain at low levels due to limited human mobility and contacts. In addition, these areas may be infrequently visited by foreign travelers. Such areas may benefit significantly from travel suspension. In some studies [54,55], beneficial delays in epidemic establishment have been reported, as a result of blocking imported cases. Apart from travel restrictions, there are other public health measures such as regular hand washing, voluntary quarantine, and school closures to reduce the impact of influenza pandemic. Compared with travel restrictions, school closure is easier to implement in a community. Past influenza pandemics have shown a particular focus on disease transmission in children. School closures resulted in a positive effect proven to be effective in reducing the disease transmission during the H1N1pdm [28]. Nevertheless, while school closures and antivirals are good for transmission reduction, they may not be for buying more time in epidemic preparation. Closing schools for a long time would induce social and economical impacts, whereas closing schools for a short period of time may not be sufficient to show effects on community transmission [56]. Other social distancing measures like cancelling public gatherings or international events raise questions about which sizes of public gatherings would warrant cancelling. These factors could be considered in future research.

Several limitations are present in our study. Restrictions for inbound travel could be beneficial to the pandemic mitigation but not outbound travel restrictions. Restrictions for outbound travel could lead to a worse situation of a pandemic growth after successive local cases arise. This is because the departure frequency is more than the arrival frequency in Hong Kong (Additional file 1: Table S1), and the excess proportion of individuals are restricted to stay and infect or transmit influenza virus to others. So there are increases to the attack rates for this scenario. Nevertheless, the restrictions on outbound travel to prevent spreading to other countries is especially beneficial for those with limited resources of pandemic prevention. Outbound travel restrictions would be better imposed during the containment phase in order to prevent a global spread of pandemic virus. As our study does not incorporate the comprehensive traveling network between countries required for a global viewpoint of pandemic spread, we cannot completely determine the value of outbound travel restrictions. Moreover, we were unable to quantify the infection risk for outbound susceptible travelers during their trip abroad because of limited information regarding their contact patterns. Although outbound passengers may become infected during their time abroad, they have nonetheless escaped from local infections. Our estimated *R*_0_ for Hong Kong was 1.4, close to that of the global median (Additional file 1: Table S2). The similar disease transmission intensity between countries would unlikely incur large infection-risk differences between outbound and local susceptible individuals, provided that the periods of H1N1pdm in different countries are not widely spaced. In addition, all travelers are assumed to undertake a single-step journey to their destination, and no adjustment for multi-step journeys is admitted in the model. Nevertheless, previously reported reports reveal little quantitative difference between single- and multi-step travel [57]. More importantly, enforcing rigorous travel restrictions has been undoubtedly unrealistic to date, since such restrictions would substantially degrade the local economy. In 2009, [58], tourism-related activities such as accommodation services, retail trade, transport services, and food and beverage services contributed 2.6% (US$5,200 million) to Hong Kong’s Gross Domestic Product (GDP). Large travel reductions thus incur high economic loss. However, increasingly severe diseases, such as SARS and influenza A (H1N1), have entered our society within recent decades, and have affected wider age groups than have past epidemics. The emergence of a highly lethal virus is feasible in the near future. In mitigating viral pandemics, the benefit to be gained from imposing travel restrictions as an adjunct to other effective control measures must be balanced against potential economic impacts. A comprehensive cost benefit analysis will thus be addressed in our future research.

## Conclusions

Our study suggested that air travel restrictions should be priorities for consideration when a new influenza pandemic begins overseas. When the pandemic is initiated in China or other places where there is land travel to Hong Kong, land travel restrictions should also be a priority. If restrictions are able to cover 99% travelers with the use of antiviral and hospitalization, the resulting pandemic delays are possible to allow vaccine production; if the restrictions cannot cover 90% or more travelers, then the peak time will happen sooner. In this case, control measures such as antiviral should be enacted earlier to alleviate the epidemic growth. To date travel restrictions have yet to gain widespread social acceptance, but the benefits may significantly outweigh the costs, especially when a new and highly intrusive virus emerges.

## Competing interests

The authors declare that they have no competing interests.

## Authors’ contributions

Both authors contributed to the study and performed statistical analysis. They all drafted the manuscript and approved the final version.

## Pre-publication history

The pre-publication history for this paper can be accessed here:

http://www.biomedcentral.com/1471-2334/12/309/prepub

## Supplementary Material

Additional file 1 **Technical appendix.** Mathematical model formulation, impact of other variations, and sensitivity analysis. Click here for file
