# Supplementary material for: Modeling the impact of air, sea, and land travel restrictions supplemented by other interventions on the emergence of a new influenza pandemic virus
Source: BMC Infect Dis. 2012 Nov 19;12:309. doi: 10.1186/1471-2334-12-309 (PMC3577649; doi:10.1186/1471-2334-12-309)
Supplement: Additional file 1 — Technical appendix. Mathematical model formulation, impact of other variations, and sensitivity analysis. [file 1471-2334-12-309-S1.pdf]

Modeling the impact of air, sea, and land travel restrictions supplemented by other interventions on the emergence of a new influenza pandemic virus

Technical Appendix

1. Mathematical model formulation
2. Impacts of other variations
   - R_0 from non-local countries;
   - Screening sensitivity at entry border points;
   - Implementation date on travel restrictions
3. Sensitivity analysis
   - Time step of model;
   - Multivariate sensitivity analysis

1 Mathematical model formulation

Basic stochastic $SEIR$ model

The model applied the concept of binomial chain process [1, 2] and the similar notation as Lekone (2006) [3]. Let Δt be a time step and $(t, t + \Delta t]$ be a time interval, we denote $S(t)$, $E(t)$, $I(t)$, and $R(t)$ as the number of individuals in **S**usceptible, **E**xposed, **I**nfected, and **R**ecovered compartments at time t, respectively. Suppose $B(t)$ is the incidence, the number of susceptible getting infected and $C(t)$ is the number of infected individuals who start to be infectious at time t. And $D(t)$ is the number of individuals recovered or died

from infectious state at time t. Suppose the population is homogeneously mixed, the system of general SEIR stochastic model with no intervention is

$$\begin{aligned} S(t+\Delta t) &= S(t) - B(t) \\ E(t+\Delta t) &= E(t) + B(t) - C(t) \\ I(t+\Delta t) &= I(t) + C(t) - D(t) \\ R(t+\Delta t) &= R(t) + D(t) \end{aligned} \tag{1}$$

An individual would have a probability p to get into next stage which follows a bernoulli distribution. So given n individuals, the number of individuals who get into next stage would follow a binomial distribution with probability m. We take $\text{bin}(m, n)$ as a binomial distribution with parameters probability m and number of total individuals n. The corresponding distributions for the classes

$$\begin{aligned} B(t) &\sim bin(1 - exp[-\frac{\beta}{N} I(t)\Delta t], S(t)) \\ C(t) &\sim bin(1 - exp(-\alpha \Delta t), E(t)) \\ D(t) &\sim bin(1 - exp(-\gamma \Delta t), I(t)) \end{aligned} \tag{2}$$

where the rate of infection is equal to $\beta I(t)/N$ for a time step, β is the transmission rate, and N is the population size. The α and γ are the constant transition rates from latent state to infectious state and from infectious state to removed state respectively. And the rates are transformed into probabilities assuming in poisson process.

Arrived and departed cases

Suppose the probabilities of travel are the same for all individuals and the probability of travelers imported from country i-th ($i = 1, 2, ..., p$) are represented by $m^I_{k,i}$ by means of transport k ($k = 1, 2, 3$) for air, sea, and land respectively. The daily probability of travel $m^I_{k,i}$ for an individual is calculated by [total frequency of travel by transport k in a year/(365 days $\times$ population N_i)]. The arrival statistics listed in Table 1 are adopted from the Hong Kong Tourism Board. We used the most up-to-date available statistics (i.e data in 2007) [4]. The statistics include the total number of visitor arrivals by countries with the mode of transports: air, sea, and land.

Here are the model compartments of imported cases in latent status,

$$IM^E_k(t) = \sum_{i=1}^{p} bin(m^I_{k,i}, E_i(t)) \tag{3}$$

and infectious status,

$$IM^I_k(t) = \sum_{i=1}^{p} bin(m^I_{k,i}, I_i(t)) \tag{4}$$

The number of latent subjects, $E_i(t)$, and the number of infectious subjects, $I_i(t)$, at time t of country i-th are generated from discrete-time $SEIR$ model based on the reproduction numbers of the countries,

Table 1. Frequency of departures and arrivals by countries with the modes of transports in 2007

		Mode of transport		
**Country**	**Total**	**Air**	**Sea**	**Land**
**Departure**				
Hong Kong	80,682,000	6,141,000	8,871,000	65,670,000
**Arrival**				
United States	1,230,927	724,023	191,178	315,726
Canada	395,167	219,469	59,004	116,694
Honduras	1,662	675	225	762
Mexico	35,706	21,260	5,821	8,625
Argentina	10,515	5,690	1,805	3,020
Brazil	40,339	19,861	8,061	12,417
Venezuela	10,896	4,356	1,612	4,928
United Kingdom	601,168	448,647	68,007	84,514
Netherlands	110,816	70,592	15,712	24,512
Denmark	30,013	18,734	4,193	7,086
Finland	21,830	13,365	3,448	5,017
Norway	18,624	12,381	2,327	3,916
Sweden	49,810	30,909	7,449	11,452
Austria	24,046	14,514	4,529	5,003
Germany	234,763	149,370	38,523	46,870
Switzerland	46,870	32,529	6,561	7,780
France	231,091	135,291	41,515	54,285
Belgium	32,413	20,190	5,114	7,109
Italy	118,841	73,043	17,564	28,234
Portugal	18,639	9,419	8,199	1,021
Spain	65,131	38,460	10,757	15,914
Russia	32,858	21,256	4,314	7,288
South Africa	72,897	47,001	4,357	21,539
Bahrain	2,500	1,833	106	561
Egypt	16,361	7,764	579	8,018
Israel	63,435	38,692	9,537	15,206
Jordan	11,084	4,809	333	5,942
Kuwait	4,366	2,838	283	1,245
Saudi Arabia	19,435	13,616	787	5,032
Turkey	41,011	20,619	2,764	17,628
United Arab Emirates	11,881	9,358	615	1,908
Australia	633,599	418,760	83,173	131,666
New Zealand	117,215	82,461	10,762	23,992
Japan	1,324,336	748,478	273,334	302,524
South Korea	876,231	507,872	136,095	232,264
Indonesia	366,217	185,197	63,102	117,918
Malaysia	504,487	237,542	105,036	161,909
Philippines	552,942	365,490	70,956	116,496
Singapore	631,963	393,423	93,794	144,746
Thailand	387,219	246,732	47,800	92,687
India	317,510	178,018	33,588	105,904
Taiwan	2,238,731	1,248,228	123,793	866,710
Macau	626,103	30,547	553,682	41,874
China	15,485,789	2,069,683	1,618,643	11,797,463

$$E_i(t+\Delta t) = E_i(t) + S_i(t)[1 - exp(-\beta_i \Delta t I_i(t)/N_i)] - E_i(t)[1 - exp(-\alpha\Delta t)]$$
$$I_i(t+\Delta t) = I_i(t) + E_i(t)[1 - exp(-\alpha\Delta t)] - I_i(t)[1 - exp(-\gamma\Delta t)] \qquad (5)$$

where $1 - exp(-\beta_i \Delta t I_i(t)/N_i)$, $1 - exp(-\alpha\Delta t)$, and $1 - exp(-\gamma\Delta t)$ are the per capita probabilities of infection, becoming infectious, and becoming recovered respectively given transmission parameter β_i in population N_i. Individual transmission parameter β_i is calculated from the basic reproduction number (R_0) of country i-th. It is defined as the average number of secondary infections produced by a typical infected individual in a wholly susceptible population. In order to allow the transmission heterogeneities between non-local countries, we will estimate the reproduction numbers by the initial exponential growth rate method [5] employing two months after dates of their first onset cases (which showed in Table 2) daily surveillance data [6] [7] [8],

$$R_0 = 1 + \frac{r^2 + (\alpha+\gamma)r}{\alpha\gamma} \qquad (6)$$

where r is the initial exponential growth rate estimated by the least square fitting to the model, i.e. $logarithm$(cumulative number of cases at time t) $\propto rt$.

At the same time, a number of infected individuals will leave and carry the pathogens away from the local city. Departure statistics are collected from the Census and Statistics Department, Hong Kong [9] and are listed in Table 1. Let m_k^E be the probability of departure from local area by the mode of transport k, the compartments of exported cases

in latent status, $EX^E(t)$, and in infectious status, $EX^I(t)$, will be $\sum_{k=1}^{3} bin(m_k^E, E(t))$ and $\sum_{k=1}^{3} bin(m_k^E, I(t))$ respectively. The calculation of m_k^E is similar to that of $m_{k,i}^I$, which adapts the departure data in Table 1.

Result of estimated R_0 and corresponding confidence interval (CI)

The reproduction numbers of the forty-four non-local countries are estimated by the initial exponential growth rate method. All of the initial growth rates are fitted significantly ($p - value < 0.05$). Showed in Table 2, the R_0 range from 1.1 to 1.9.

Antiviral and hospitalization

Two new compartments are added into the model, antiviral **T**reatment $T(t)$ and **H**ospitalization $H(t)$. Once individuals become infectious, they seek for antiviral treatment and hospitalization with proportions p_T and p_H respectively. With regard to limited resources, part of them may be untreated as proportions p_U. We adapt a ψ fraction reduction of infectiousness for individuals who receive antiviral. Suppose classes $M(t)$ and $N(t)$ are the number of infectious individuals who take antiviral treatment and hospitalization at time t respectively. The $P(t)$ and $Q(t)$ are the number of removed individuals from antiviral treatment and hospitalization with transition rates γ_T and γ_H to the removed status.

Stochastic $SEIR$ model with interventions

Because infectious individuals include those being treated and hospitalized, the probability of a susceptible person becoming infected is equal to $1-exp[\beta[I(t)+(1-\psi)T(t)+H(t)]/N]$ for a time step Δt. Given ν is the sensitivity of the entry screening board, so only ν proportion of imported infectious individuals are able to be identified as positive cases

Table 2. Start date of epidemic (2009) and estimated R_0 (CI)

Country	Start date MM/DD	R_0 (CI)
United States	04/21	1.62 (1.52, 1.72)
Canada	04/28	1.42 (1.38, 1.47)
Honduras	05/23	1.39 (1.31, 1.48)
Mexico	03/11	1.56 (1.52, 1.59)
Argentina	05/09	1.81 (1.73, 1.90)
Brazil	05/09	1.45 (1.42, 1.49)
Venezuela	05/29	1.43 (1.37, 1.49)
United Kingdom	04/28	1.51 (1.47, 1.54)
Netherlands	05/01	1.42 (1.38, 1.47)
Denmark	05/02	1.37 (1.32, 1.42)
Finland	05/13	1.32 (1.30, 1.35)
Norway	05/11	1.27 (1.26, 1.28)
Sweden	05/07	1.39 (1.37, 1.41)
Austria	04/30	1.24 (1.20, 1.27)
Germany	04/30	1.37 (1.34, 1.39)
Switzerland	05/01	1.38 (1.35, 1.41)
France	05/02	1.33 (1.31, 1.35)
Belgium	05/14	1.24 (1.23, 1.26)
Italy	05/03	1.29 (1.27, 1.31)
Portugal	05/06	1.24 (1.21, 1.27)
Spain	04/28	1.30 (1.25, 1.35)
Russia	05/23	1.06 (1.04, 1.08)
South Africa	06/18	1.69 (1.62, 1.76)
Bahrain	05/27	1.35 (1.31, 1.40)
Egypt	06/03	1.35 (1.30, 1.40)
Israel	04/29	1.42 (1.39, 1.45)
Jordan	06/17	1.26 (1.23, 1.30)
Kuwait	05/25	1.10 (1.09, 1.11)
Saudi Arabia	06/03	1.48 (1.43, 1.54)
Turkey	05/17	1.30 (1.27, 1.32)
United Arab Emirates	05/25	1.30 (1.25, 1.34)
Australia	05/09	1.87 (1.77, 1.98)
New Zealand	04/29	1.35 (1.30, 1.41)
Japan	05/09	1.44 (1.35, 1.53)
South Korea	05/03	1.43 (1.39, 1.46)
Indonesia	06/24	1.69 (1.62, 1.75)
Malaysia	05/16	1.59 (1.54, 1.64)
Philippines	05/22	1.66 (1.60, 1.71)
Singapore	05/27	1.58 (1.53, 1.64)
Thailand	05/14	1.80 (1.71, 1.88)
India	05/17	1.56 (1.51, 1.60)
Taiwan	05/20	1.28 (1.23, 1.32)
Macau	06/19	1.39 (1.34, 1.45)
China	05/12	1.52 (1.50, 1.55)

and be voluntary quarantined. Let f_k be the restriction fraction for import transportation k-th, the stochastic system is as follow,

$$
\begin{aligned}
S(t+\Delta t) &= S(t) - B(t)\\
E(t+\Delta t) &= E(t) + B(t) + \sum_k (1-f_k) IM_k^E(t) - EX^E(t) - C(t)\\
I(t+\Delta t) &= I(t) + C(t) + (1-\nu)\sum_k (1-f_k) IM_k^I(t) - EX^I(t) - D(t) - M(t) - N(t)\\
T(t+\Delta t) &= T(t) + M(t) - P(t)\\
H(t+\Delta t) &= H(t) + N(t) - Q(t)\\
R(t+\Delta t) &= R(t) + D(t) + P(t) + Q(t)
\end{aligned}
\tag{7}
$$

The distributions for the classes are

$$
\begin{aligned}
B(t) &\sim bin(1 - exp[-\frac{\beta}{N}[I(t) + (1-\psi)T(t) + H(t)]\Delta t]), S(t))\\
C(t) &\sim bin(1 - exp(-\alpha\Delta t), E(t))\\
M(t) &\sim bin(p_T\Delta t, I(t))\\
N(t) &\sim bin(p_H\Delta t, I(t))\\
D(t) &\sim bin(p_U[1 - exp(-\gamma_R\Delta t)], I(t))\\
P(t) &\sim bin(1 - exp(-\gamma_T\Delta t), T(t))\\
Q(t) &\sim bin(1 - exp(-\gamma_H\Delta t), H(t))
\end{aligned}
\tag{8}
$$

The descriptions of the parameters are highlighted in Table 3. A simple schematic flow is showed in Figure 1.

Table 3. Parameters, definitions, and values for the model

Parameter	Definition	Value	Ref/remarks
R_0	Basic reproductive number	Estimated	Local baseline estimated about 1.4
$T_E = 1/\alpha$	Average latent period (days)	1.45	[11] [12]
T_I	Average infectious period (days)	2.9	[11] [12]
T_T	Average infectious period (days) for individuals treated with antiviral treatment	1.4	[13]
T_H	Average infectious period (days) for hospitalized individuals	1.4	[13]
p_T	Proportions of infectious subjects selected for treatment	0.12	[14]
p_H	Proportions of infectious subjects selected for hospitalization	0.06	[15]
p_U	Proportions of untreated infectious subjects	$1 - p_T - p_H$	
γ_R	Transition rates from infectious state to removed state	$1/T_I$	
γ_T	Transition rates from treatment state to removed state	$1/T_T$	
γ_H	Transition rates from hospitalization state to removed state	$1/T_H$	
f_k	Restriction fraction for k-th transportation	90%, 99%	Assumption
ψ	Fraction of infectiousness reduction for antiviral treatment	60%	[16]
ν	Sensitivity of the screening board for infectious subjects	0.3	[10]

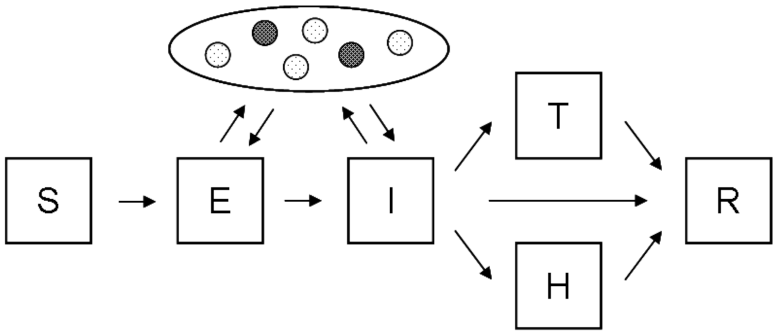

Figure 1. Schematic flow of $SEIR$ model which incorporated the compartments treatment and hospitalization with import-export latent and infectious individuals

Epidemic evolution

The pandemic is seeded according to the start dates (Table 2) of each country [6] [7]. The earliest epidemic was seeded in Mexico on March 11, 2009 [8]. Each country will develop its own infected cases by generating from the discretized-time $SEIR$ model based on the estimated reproduction number. At the same time, the countries will send their infected cases to Hong Kong and the local epidemic evolution will be initiated by the successive imported cases via air, sea, and land traffic. The first passage times (FPT), first one hundred passages times (FHPT), and the peak time are calculated for different restriction strategies.

Baseline scenario

Since the Hong Kong Government confirmed the first imported case of H1N1pdm on May 1, 2009 [17], the parameters $E_i(0)$ and $I_i(0)$ are roughly assumed the same for all countries and are iteratively estimated, thereby minimizing the difference between the reported

date and the simulated first passage time (FPT). Allowing for stochastic variability, the baseline transmission rate (β) is fitted using the local surveillance data for the first two months following the day of the first local import, in the absence of travel restrictions and intervention. Local daily surveillance of confirmed infected cases (Figure 2) was available from press releases on human swine flu, published by the Department of Health, Hong Kong [18]. Optimum parameter is chosen which had average minimum relative mean square error between empirical and estimated cumulative incidence by Monte Carlo simulation. The reproduction number is the product of the transmission rate and the average infectious period. We adapt the range of parameter space for the reproduction numbers according to previous influenza A (H1N1) studies [8] [11] [19] [20].

Result of baseline estimation

The estimated parameters $E_i(0)$ and $I_i(0)$ are equal to 90 individuals which obtain May 4 as a mean FPT with a 95% confidence interval [Apr 14, May 16]. The local estimated R_0 is about 1.4 during the first two months after the reported FPT. The fitted cumulative incidence curve from data was showed in Figure 2.

Computer simulation

The model is implemented in software SAS 9.1.3. Simulation is started by the first global onset case with one day time step. The program generates one hundred realizations for each scenario. The medians, means, and the 95% non-parametric confidence intervals of the incidence, peak times, and the time of imported case arrivals are calculated over the realizations among different scenarios.

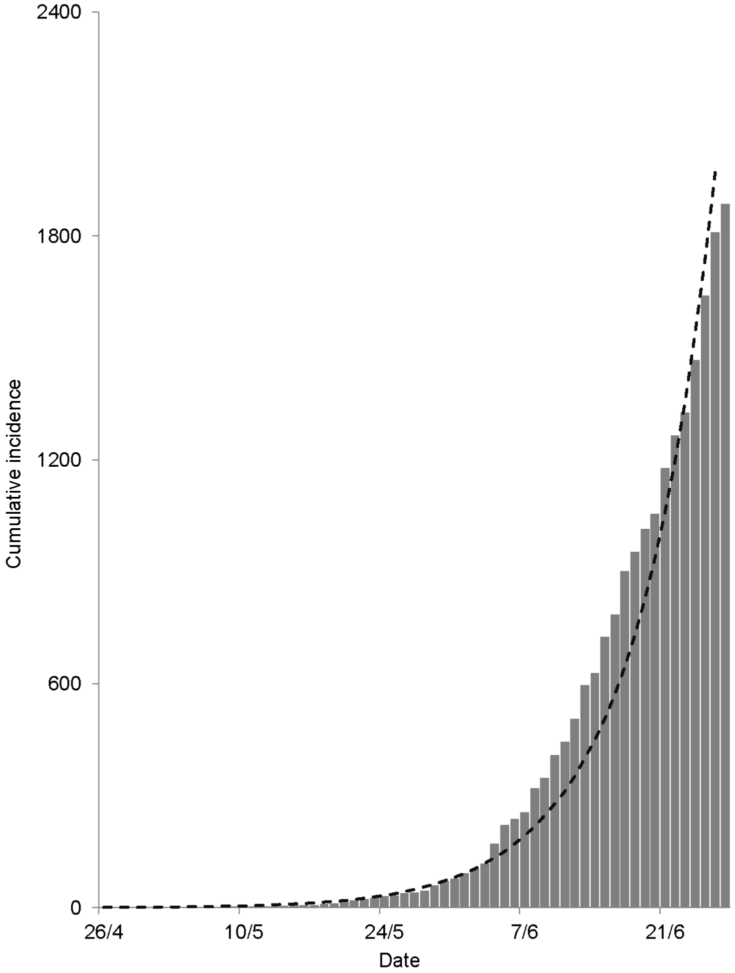

Figure 2. Daily confirmed cases in Hong Kong and the median curve simulated from the best fitted model. The confirmed cumulative cases (the grey bar in the figure) is from the press releases on human swine flu, published by the Department of Health, Hong Kong. The median cumulative cases (the dash line) were simulated by 100 realizations from the best fitted model

2 Impact of other variations

Variations of R_0 from non-local countries

We varied the R_0s from 44 foreign countries by 20%, in order to test these effects on our results. The increased and decreased R_0s ranged from 1.3 to 2.2 (median 1.7), and from 0.8 to 1.5 (median 1.1), respectively. Although five countries did not occur any outbreak i.e. $R_0 < 1$, it made small impact on the size of infected cases exportation among all countries.

Showed in Figure 3, the external travel restrictions performed slightly better in deferring the FPTs and the FHPTs when the R_0s from non-local countries decreased. Given the R_0s increased by 20%, the medians of FPT and the FHPT were day 44-th and day 74-th respectively with no travel restriction; the medians of FPT and the FHPT were day 63-th and day 112-th respectively when the R_0s decreased 20%. Amongst all situations for the changes of the R_0s, either 90% or 99% of air travel rescaling could have about 1 week delay for the FPTs; but once all means of transport were 90% or 99% restricted, the FPT would have one month more delay when the R_0s decreased 20% compared to that of the R_0s with 20% increases. Moreover, the FHPT could be delayed for more than 2.5 months with 20% decreases of the R_0s, whereas the FHPT was delayed for 1.5 months with 20% increases of the R_0s for a 99% restriction of all means of transport.

Since the number of imported cases depended on the changes of the R_0 from the non-local countries, the growth of the local epidemic was affected by the cases passage times (Figure 4). When the R_0s increased by 20%, the five months' cumulative AR attained 19% and the epidemic ended at the seventh month since the first global case arose. During the first five months, the blockings of all external means of transport were still effective on controlling the cumulative ARs. A 99% travel restriction maintained about 12% of seven

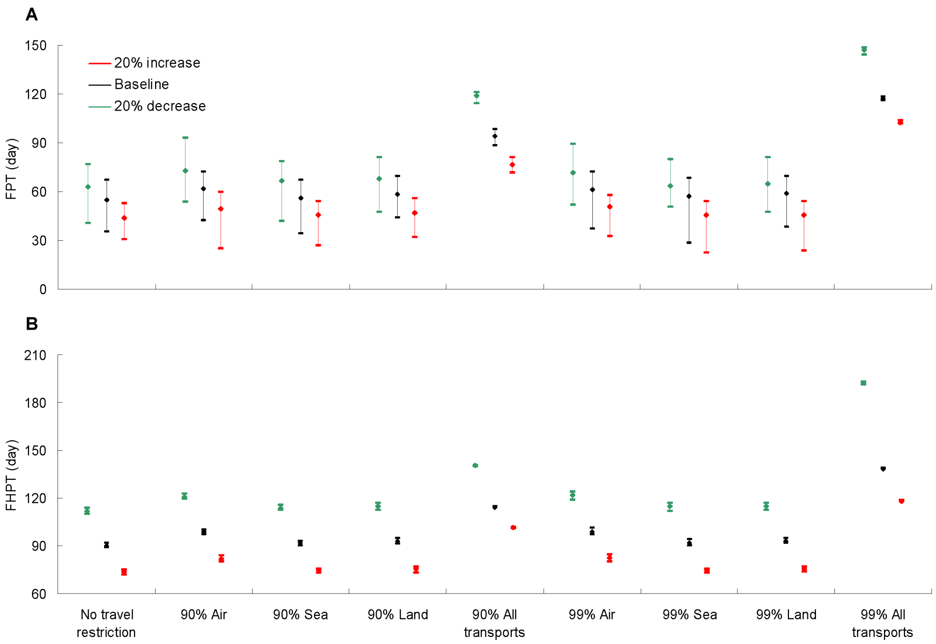

Figure 3. FPT and FHPT when non-local countries R_0s increased by 20% or decreased by 20%. The upper panel (A) and the lower panel (B) illustrate the FPT and the FHPT respectively. Day one was taken to be March 11, 2009 (the time of the first global case onset). The medians are demonstrated as the dots in the interpolations; the corresponding lower and upper bounds of the 95% non-parametric confidence intervals are demonstrated as the lower cups and upper cups respectively.

months' cumulative AR (Figure 4A). Similar to the baseline scenario, the travel restriction made greater impacts on slowing down the ARs increase with the use of antiviral and hospitalization (AH); a 99% rescaling of means of all transport controlled the final AR at about 20% in addition to the use of AH (Figure 4B). When the R_0s decreased by 20%, the travel restrictions performed better in slowing down the disease transmission. Even if only the air travel was either 90% or 99% restricted, the seven months' cumulative ARs would have reduced about 15% compared to that of no intervention (Figure 4C). A 99% restriction of all means of transport would have halted the local spread i.e. cumulative ARs $< 0.1\%$ in seven months' time whether or not the AH had been used (Figure 4C and 4D). However, the final cumulative ARs would not be affected by the changes of the R_0s from non-local countries.

Variations of screening sensitivity at entry border points

In the baseline scenarios, we set the screening sensitivity at entry border points as 30%; here, we assess the model's output at extremely high (95%) and low (5%) screening sensitivities. According to Figure 5 and 6, the screening sensitivities at entry border points affected slightly on the times of cases arrival. Amongst most of the travel restriction strategies, a 95% screening sensitivity showed at most one to two weeks additional delay to the FHPTs compared to that of a 5% screening sensitivity (Figure 5).

The increase of the screening sensitivity at entry border points offered a moderate benefit on slowing down the growths of cumulative ARs. Showed in Figure 6A-D, a 95% screening sensitivity showed only half of five months' cumulative ARs compared to that of a 5% screening sensitivity. The 95% screening sensitivity also decreased the seven months' cumulative ARs by about 10% in most of the restriction strategies whether or not the AH had been imposed.

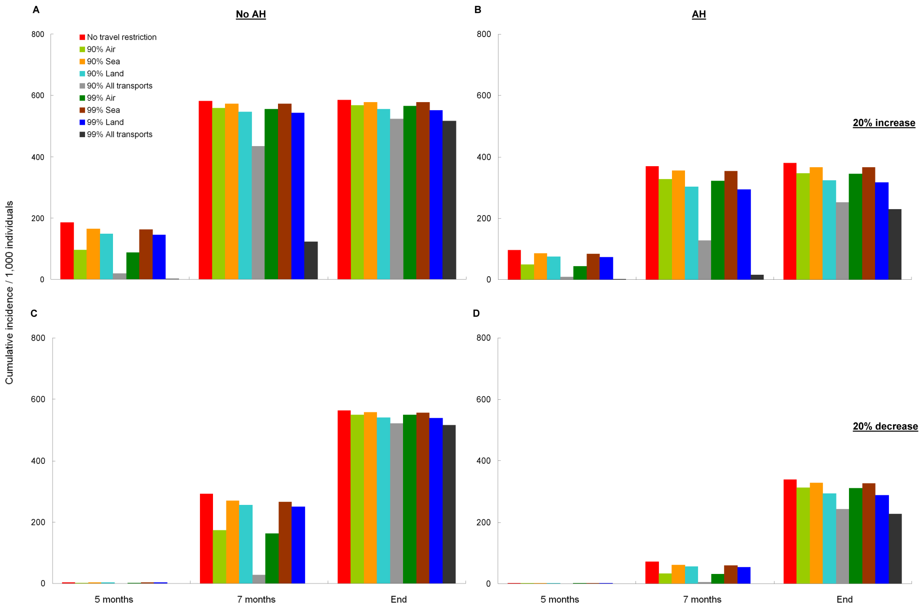

Figure 4. Median cumulative ARs on different time points when non-local countries R_0s increased by 20% or decreased by 20%. The upper panel (A and B) and the lower panel (C and D) show the cumulative ARs with the non-local countries' R_0s increased by 20% and decreased by 20% respectively. The absences and the presences of the uses of the antiviral and hospitalization are illustrated in left-hand column (A and C) and right-hand column (B and D) respectively. The baseline scenario ($R_0 = 1.4$) was adopted.

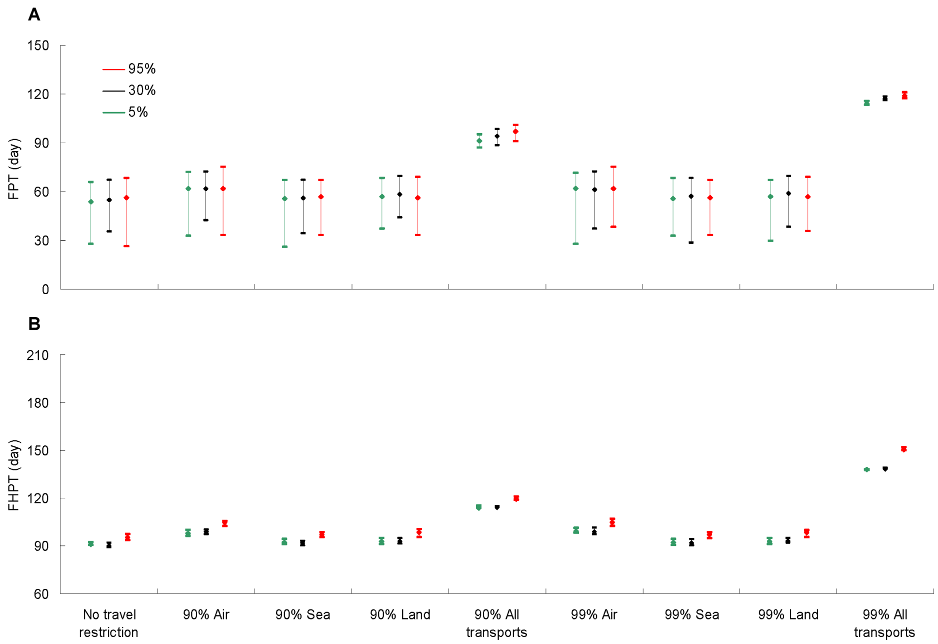

Figure 5. FPT and FHPT when screening sensitivity increased to 95% or decreased to 5%. The upper panel (A) and the lower panel (B) illustrate the FPT and the FHPT respectively. Day one was taken to be March 11, 2009 (the time of the first global case onset). The medians are demonstrated as the dots in the interpolations; the corresponding lower and upper bounds of the 95% non-parametric confidence intervals are demonstrated as the lower cups and upper cups respectively.

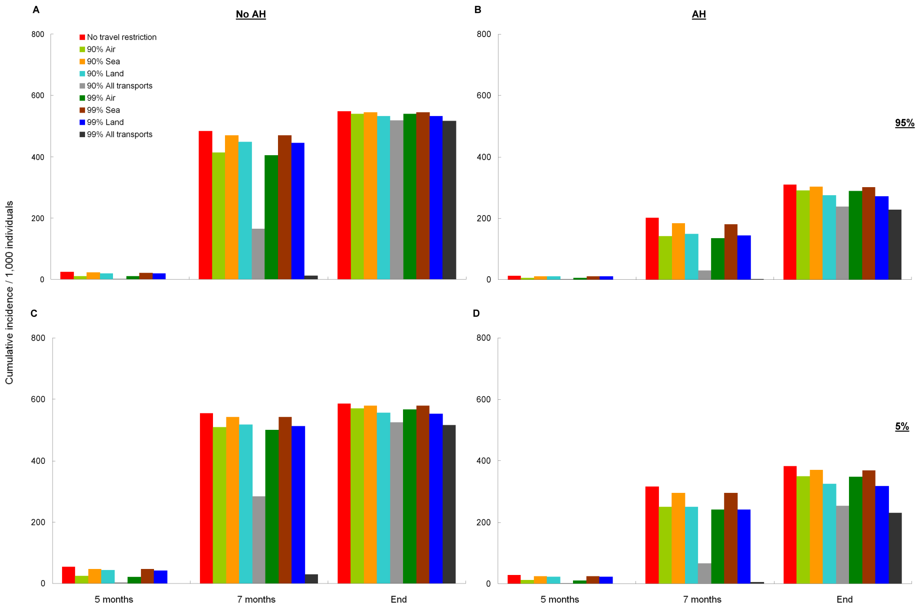

Figure 6. Median cumulative ARs on different time points when screening sensitivity increased to 95% or decreased to 5%. The upper panel (A and B) and the lower panel (C and D) show the cumulative ARs with the screening sensitivities increased to 95% and decreased to 5% respectively. The absences and the presences of the uses of the antiviral and hospitalization are illustrated in left-hand column (A and C) and right-hand column (B and D) respectively. The baseline scenario ($R_0 = 1.4$) was adopted.

Variations of implementation date on travel restrictions

We tested the impact of delaying the imposition of travel restrictions to five and three months following the first global import. Showed in Figure 7A and 7B, imposing travel restrictions five months after the first global case arose would be too late obviously. Even if all means of transport had been 99% rescaled, the reduction in the cumulative AR was too small. However, it could still decrease the seven months' cumulative AR by no more than 10% if the growth of the epidemic was slowed down by the use of AH. Showed in Figure 7C, imposing the travel restrictions three months after the first global case arose would be a little bit late; but fractional blockings on all means of transport worked well in deferring the growth of the ARs. The 99% restriction would reduced the five months' and seven months' cumulative ARs more than half of that without intervention. With the use of AH, imposing the 99% restriction of all mean of transport was able to control the cumulative AR by no more than 2% in the first seven months; a 90% restriction could still maintain the average seven months' cumulative AR about 6% to 7% (Figure 7D).

3 Sensitivity analysis

Time step of model

The simulation results were based on the stochastic models with a time step of $\Delta t = 1$ day. The simulations were repeated with $\Delta t = 0.5$ day. Figure 8 showed the results for comparison. According to the results, the incidence growth curves differed moderately compared with that of $\Delta t = 1$ day (main text, Figure 3); the daily ARs were less severe for scenarios with $\Delta t = 0.5$ day. However, there were only slight differences for the impacts of interventions on the baseline scenario between two kinds of time-step settings. For

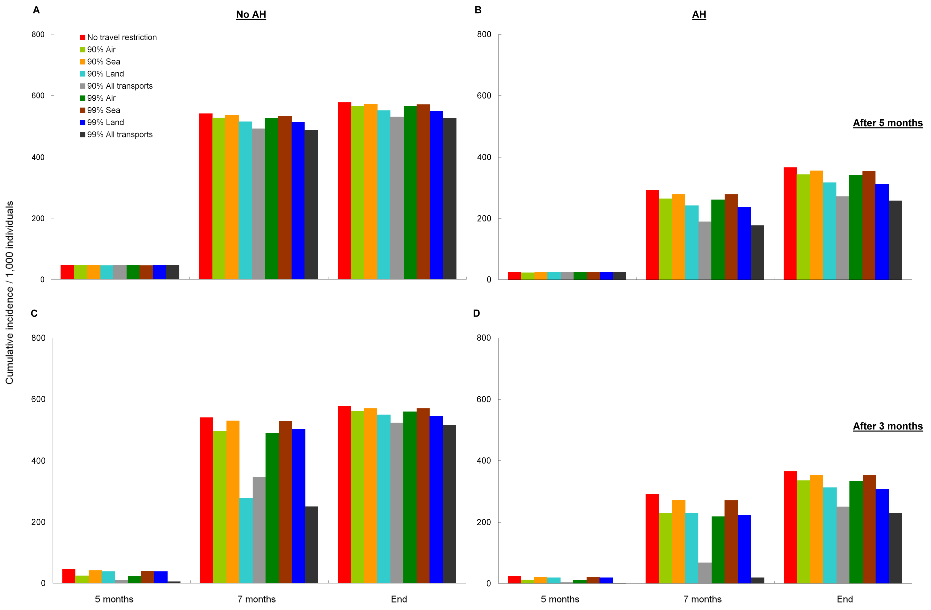

Figure 7. Median cumulative ARs on different time points when implementation date of travel restrictions delayed for five months or three months. The upper panel (A and B) and the lower panel (C and D) show the cumulative ARs with the implementation dates on travel restrictions delayed for five months and three months respectively. The absences and the presences of the uses of the antiviral and hospitalization are illustrated in left-hand column (A and C) and right-hand column (B and D) respectively. The baseline scenario ($R_0 = 1.4$) was adopted.

example, a 99% restriction of all transport modes deferred the peak for about 12 weeks (Figure 8B) in both time-step settings. Models with either $\Delta t = 0.5$ day or $\Delta t = 1$ day drew similar conclusions in the study.

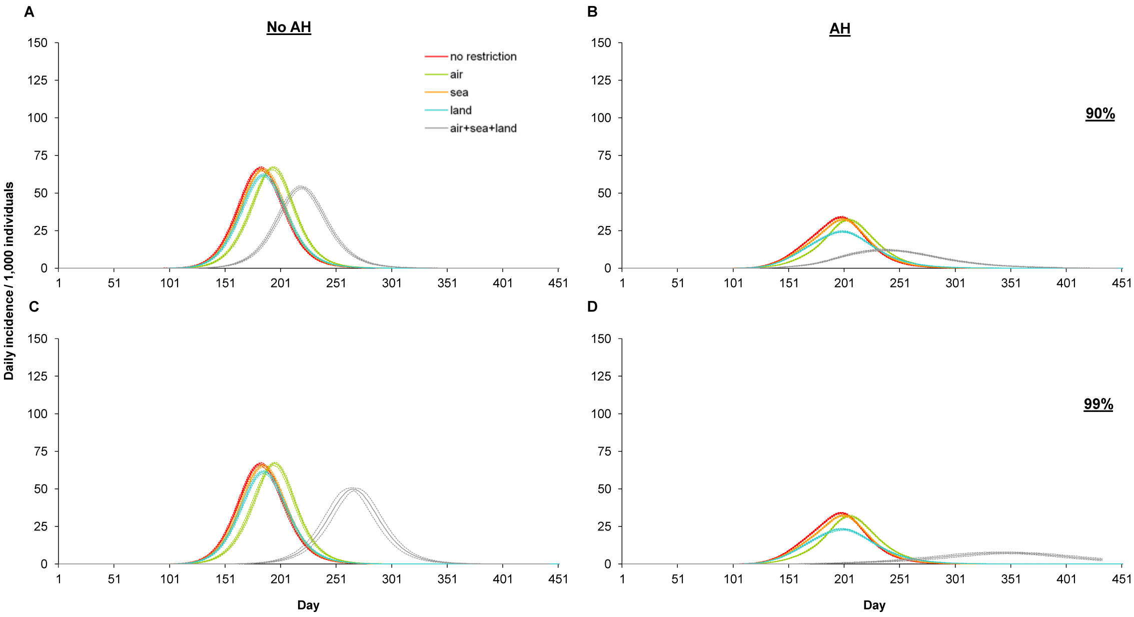

Figure 8. Daily incidences at the baseline scenario given $\Delta t = 0.5$ day. The absences and the presences of the uses of antiviral and hospitalization are illustrated in the left-hand column (A and C) and in the right-hand column (B and D), respectively. The upper panel (A and B) and the lower panel (C and D) illustrate the 90% and the 99% restriction rescaling, respectively. Day one was taken to be March 11, 2009 (the time of the first global case onset). The solid lines represent the average cases; the dotted lines represent the corresponding lower and upper bounds of the 95% non-parametric confidence intervals; AH = antiviral and hospitalization.

Multivariate sensitivity analysis

A multivariate sensitivity analysis which varied the following parameters with their prior distributions:

- Length of latent period (days) $\sim$ Uniform(range from 1 to 2)

- Sensitivity of the screening board for infectious subjects ∼ Uniform(range from 0.05 to 0.5)

- Length of infectious period reduction (days) by taking antivirals ∼ Uniform(range from 1 to 2)

- Length of infectious period reduction (days) by hospitalization ∼ Uniform(range from 1 to 2)

- Fraction of infectiousness reduction for antiviral treatment ∼ Uniform(range from 0.3 to 0.9)

was performed. Each random set of parameters was simulated before every realization.

Figures 9 and 10 showed the results. The incidence curves were moderately sensitive to the variations of parameters. Restrictions on a single mode of transport had less apparent impact due to moderate deviations. The range of peak times from imposing 99% restriction on all modes of transports was wider. However, the central tendency of the intervention effects were quite stable. The impacts of interventions on the baseline scenario did not differ much compared with the study main findings. For example, when a 99% restriction of all transports was imposed, the peak was averagely deferred to the ninth month and the eleventh month respectively in absences and presences of the uses of antiviral and hospitalization.

References

1. Greenwood M (1931) On the Statistical Measure of Infectiousness. J Hyg (Lond) 31: 336-351.

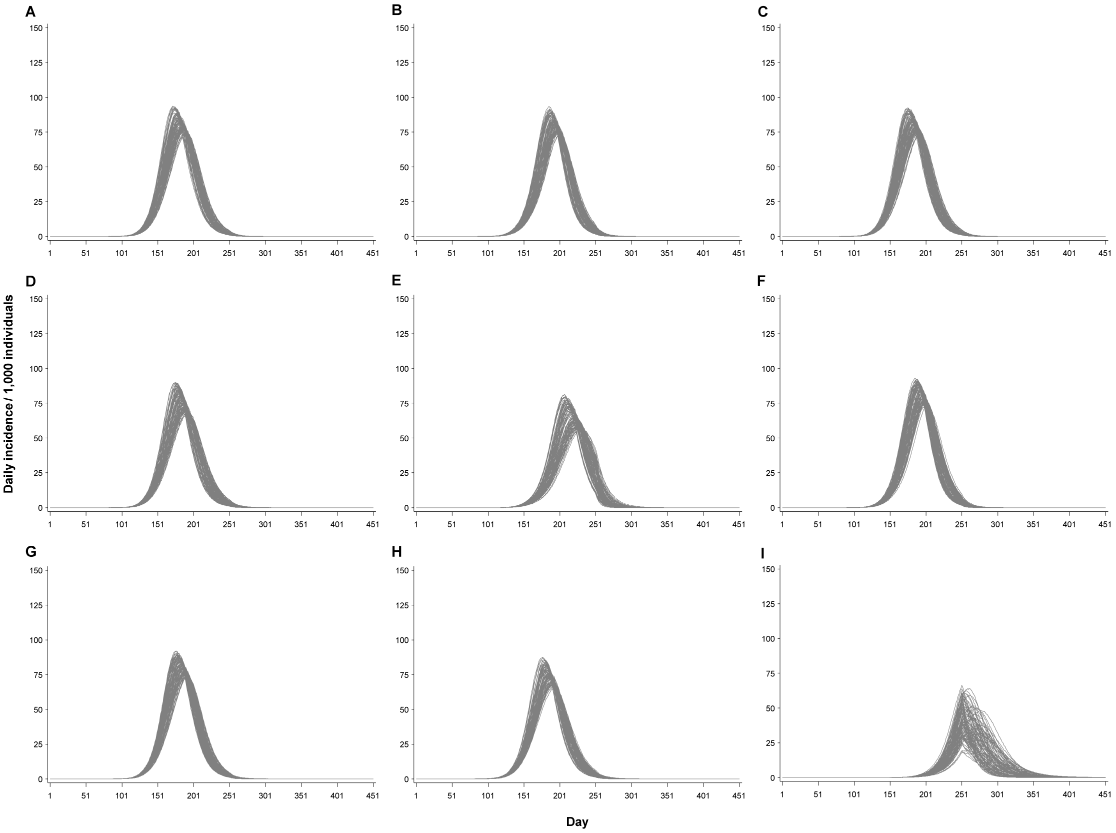

Figure 9. Daily incidences in absences of the uses of antiviral and hospitalization for a multivariate sensitivity analysis. One hundred simulated curves were drawn in each figure: (A) No travel restrictions. (B) 90% air travel restrictions. (C) 90% sea travel restrictions. (D) 90% land travel restrictions. (E) 90% all travel restrictions. (F) 99% air travel restrictions. (G) 99% sea travel restrictions. (H) 99% land travel restrictions. (I) 99% all travel restrictions.

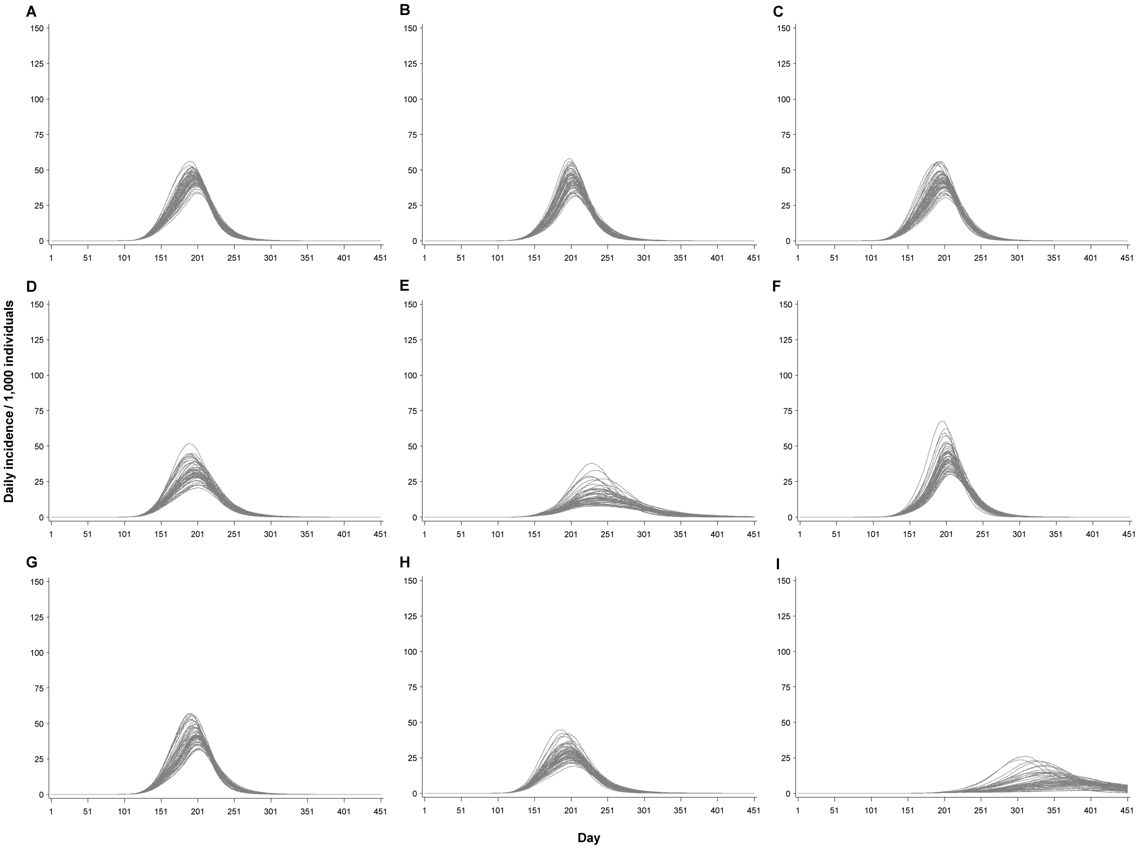

Figure 10. Daily incidences in presences of the uses of antiviral and hospitalization for a multivariate sensitivity analysis. One hundred simulated curves were drawn in each figure: (A) No travel restrictions. (B) 90% air travel restrictions. (C) 90% sea travel restrictions. (D) 90% land travel restrictions. (E) 90% all travel restrictions. (F) 99% air travel restrictions. (G) 99% sea travel restrictions. (H) 99% land travel restrictions. (I) 99% all travel restrictions.

2. Abbey H (1952) An examination of the Reed-Frost theory of epidemics. Hum Biol 24: 201-33.

3. Lekone, Phenyo E, Finkenstadt, Barbel F (2006) Statistical inference in a stochastic epidemic SEIR model with control intervention: Ebola as a case study. Biometrics 62: 1170-1177.

4. Hong Kong Tourism Board (2007) Visitor arrival statistics. Available: http://partnernet.hktourismboard.com/pnweb/jsp/doc/listdocl.jsp?charset=en&doc_id=107560&filename=vas+12200. Accessed 2010 Apr 19.

5. Chowell G, Hengartnerb N, Castillo-Chaveza C, Fenimorea P, Hyman J (2004) The basic reproductive number of Ebola and the effects of public health measures: The cases of Congo and Uganda. J of Theor Bio 229: 119-126.

6. World Health Organization. (2009) Situation updates - Pandemic (H1N1) 2009. Available: http://www.who.int/csr/disease/swineflu/updates/en/index.html. Accessed 2010 Jul 30.

7. European Centre for Disease Prevention and Control (2009) Daily update on the 2009 influenza A (H1N1) pandemic. Available: http://www.ecdc.europa.eu/en/healthtopics/h1n1/pages/daily_update.aspx. Accessed 2009 Dec 20.

8. Fraser C, Donnelly CA, Cauchemez S, Hanage WP, Van Kerkhove MD, et al. (2009) Pandemic potential of a strain of Influenza A (H1N1): Early findings. Science 324: 1557-1561.

9. Census and Statistics Department, HKSAR (2010) Transport, communications and tourism statistics. Hong Kong resident departures by control point. Avail-

able: http://www.censtatd.gov.hk/filemanager/en/content_807/transport.pdf. Accessed 2009 Nov 25.

10. Cowling B, Lau L, Wu P, Wong H, Fang V, et al. (2010) Entry screening to delay local transmission of 2009 pandemic influenza A (H1N1). BMC Infect Dis 10: 82.

11. Flahault A, Vergu E, Boelle PY (2009) Potential for a global dynamic of Influenza A (H1N1). BMC Infect Dis 9: 129.

12. Boelle P, Bernillon P, Desenclos J (2009) A preliminary estimation of the reproduction ratio for new influenza A(H1N1) from the outbreak in Mexico, March-April 2009. Euro Surveill 14: 19205.

13. Centers for Disease Control and Prevention (2010) Questions and answers: Antiviral drugs, 2009-2010 flu season. Available: http://www.cdc.gov/h1n1flu/antiviral.htm/. Accessed 2010 Apr 19.

14. Gani R, Hughes H, Fleming D, Griffin T, Medlock J, et al. (2005) Potential impact of antiviral drug use during influenza pandemic. Emerg Infect Dis 11: 1355-1362.

15. Wu JT, Riley S, Fraser C, Leung GM (2006) Reducing the impact of the next influenza pandemic using household-based public health interventions. PLoS Med 3: e361.

16. Ferguson NM, Cummings DAT, Fraser C, Cajka JC, Cooley PC, et al. (2006) Strategies for mitigating an influenza pandemic. Nature 442: 448-452.

17. Hong Kong Information Services Department. News Archives. HKSAR Press Releases on May 2, 2009. Press releases on human swine flu. Available: http://www.isd.gov.hk/pr/eng/. Accessed 2010 Jan 28.

18. Hong Kong Information Services Department (2009) News Archives. HKSAR Press Releases from 2009 May 1 to 2009 Dec 31. Press releases on human swine flu. Available: http://www.isd.gov.hk/pr/eng/. Accessed 2010 Apr 19.

19. Brouwers L, Cakici B, Camitz M, Tegnell A, Boman M (2009) Economic consequences to society of pandemic H1N1 influenza 2009: Preliminary results for Sweden. Euro Surveill 14: 19333.

20. De Silva U, Warachit J, Waicharoen S, Chittaganpitch M (2009) A preliminary analysis of the epidemiology of influenza A(H1N1)v virus infection in Thailand from early outbreak data, June-July 2009. Euro Surveill 14: 19292.

21. Lessler J, Reich NG, Brookmeyer R, Perl TM, Nelson KE, et al. (2009) Incubation periods of acute respiratory viral infections: a systematic review. Lancet Infect Dis 9: 291-300.
